# Linking connectivity of deep brain stimulation of nucleus accumbens area with clinical depression improvements: a retrospective longitudinal case series

**DOI:** 10.1007/s00406-023-01683-x

**Published:** 2023-09-05

**Authors:** Simona Leserri, Alba Segura-Amil, Andreas Nowacki, Ines Debove, Katrin Petermann, Lea Schäppi, Maria Giulia Preti, Dimitri Van De Ville, Claudio Pollo, Sebastian Walther, T. A. Khoa Nguyen

**Affiliations:** 1 Department of Neurosurgery, Inselspital, Bern University Hospital, University of Bern, Bern, Switzerland; 2 ARTORG Center for Biomedical Engineering Research, University Bern, Bern, Switzerland; 3 Neuro-X Institute, École Polytechnique Fédérale de Lausanne (EPFL), Lausanne, Switzerland; 4 Department of Neurology, Inselspital, Bern University Hospital, University of Bern, Bern, Switzerland; 5 Translational Research Center, University Hospital of Psychiatry and Psychotherapy, University of Bern, Bern, Switzerland; 6 CIBM Center for Biomedical Imaging, Lausanne, Switzerland; 7 Department of Radiology and Medical InformaticsFaculty of Medicine, University of Geneva, Geneva, Switzerland; 8 ARTORG IGT, Murtenstrasse 50, 3008 Bern, Switzerland

**Keywords:** Deep brain stimulation, Treatment-resistant depression, Nucleus accumbens area, Connectivity, Depression networks, Subgenual anterior cingulate cortex

## Abstract

Treatment-resistant depression is a severe form of major depressive disorder and deep brain stimulation is currently an investigational treatment. The stimulation’s therapeutic effect may be explained through the functional and structural connectivities between the stimulated area and other brain regions, or to depression-associated networks. In this longitudinal, retrospective study, four female patients with treatment-resistant depression were implanted for stimulation in the nucleus accumbens area at our center. We analyzed the structural and functional connectivity of the stimulation area: the structural connectivity was investigated with probabilistic tractography; the functional connectivity was estimated by combining patient-specific stimulation volumes and a normative functional connectome. These structural and functional connectivity *profiles* were then related to four clinical outcome scores. At 1-year follow-up, the remission rate was 66%. We observed a consistent structural connectivity to Brodmann area 25 in the patient with the longest remission phase. The functional connectivity analysis resulted in patient-specific *R-maps* describing brain areas significantly correlated with symptom improvement in this patient, notably the prefrontal cortex. But the connectivity analysis was mixed across patients, calling for confirmation in a larger cohort and over longer time periods.

**Supplementary Information:**

The online version contains supplementary material available at 10.1007/s00406-023-01683-x.

## Introduction

Major depressive disorder (MDD) is characterized by depressed mood or loss of interest, and often presents a chronic course [1]. About a third [2] of affected patients do not respond adequately to conventional pharmaceutical or cognitive behavioral therapy [3, 4] and develop treatment-resistant depression (TRD), a particularly debilitating subcategory of MDD [5].

Depressive symptoms have been associated with pathological connectivity in at least four brain subnetworks: the ventral limbic affective network, the dorsal cognitive control network, the default mode network, and the frontal–striatal reward network [6–8]. The dysregulation of the reward network has been specifically related to anhedonia (i.e., loss of interest, motivation, and pleasure) [6, 9–12].

Deep brain stimulation (DBS) is being investigated as treatment option for TRD patients [10, 13–15]. The antidepressant effect of DBS depends on the neuroanatomical target, the stimulation parameters, and the modulation of both the target and the connected networks [16]. Several stimulation targets have been proposed: the subcallosal cingulate gyrus [13], the medial forebrain bundle [17], the ventral capsule/ventral striatum or anterior limb of internal capsule, the lateral habenula, the inferior thalamic peduncle, and the nucleus accumbens (NAc) [16, 18].

These targets presumably improve depressive symptoms by modulating a common network [19]. A recent study [20] suggests that not only different DBS targets, but also transcranial magnetic stimulation targets and brain lesions are interconnected and converge on a common depression network. Likewise, different DBS targets for obsessive–compulsive disorder, a disorder closely related to TRD [17, 21], converge on one network [22–24]. Still, it is essential to differentiate the impact of each DBS target and to analyze longitudinal outcome data to inform more effective DBS for TRD [25, 26].

The NAc has been investigated as a target because of its central role in the reward circuitry [27]. Structurally, the medial forebrain bundle connects the NAc to the ventral tegmental area, the ventromedial hypothalamus, the lateral hypothalamus, and the amygdala, with convergence onto the prefrontal cortex [28, 29], all crucial regions in the pathobiology of depression [6]. In particular, microstructural white-matter alterations of the medial forebrain bundle have been associated with symptoms of anhedonia [11]. Functionally, the serotonin–dopamine–glutamate interactions occurring in the NAc also make it a candidate target for DBS for MDD. Moreover, modulation of this area might restore the equilibrium between inhibitory and excitatory neurotransmission necessary for reward processing and mood regulation [27].

NAc gray-matter alterations have been associated with MDD symptoms and lifetime occurrence of the disease [30]. Additionally, reduced responses to rewarding stimuli in the NAc have been reported in patients with MDD [27, 31]; and anhedonia has been correlated with functional connectivity of specific NAc subregions [32]. Finally, clinical and neurobiological studies demonstrated that DBS of the NAc area reduces anhedonia [14, 33, 34].

The objective of the current study was to correlate clinical improvement related to DBS of the NAc area with the area’s structural and functional connectivity, measured through diffusion MRI (dMRI) and functional MRI (fMRI). We hypothesized a correlation between DBS connectivity to the reward network and clinical improvement. Furthermore, we expected beneficial NAc area DBS to be functionally and/or structurally connected to other nodes of the depression network.

## Materials and methods

### Patients and data

Four patients with TRD from the University Hospital Bern (Bern, Switzerland) were initially included in this retrospective study. The local ethics committee approved the study (2020-02392), and patient consent was obtained prior to the treatment. After DBS implantation, the patients agreed to have their data used in this post hoc analysis. Such consent was given to the neurology department during their standard pre-surgical workup. Patients 1, 3, and 4 were diagnosed with recurrent MDD, while patient 2 had bipolar disorder with predominantly depressive episodes. None of the patients had medical or neurological comorbidities. All underwent cycles of standard antidepressants (more than three, including tricyclics), lithium, ketamine, specific psychotherapy interventions for TRD [cognitive behavioral therapy and cognitive behavioral analysis system of psychotherapy (CBASP)], and electro-convulsive therapy prior to surgery, yet failed to reach remission. The study participants were the only patients who received bilateral DBS surgery for depression at our center between 2017 and 2020.

Targeting of the NAc area was performed on structural MRI and dMRI sequences with tractography of the medial forebrain bundle. The target was placed just posterior to the NAc in order to include the afferent fibers of the medial forebrain bundle before reaching the anterior limb of the internal capsule (Supplementary Fig. 1). Table 1 reports the diagnosis, sex, age, and baseline depression scores of the patients at the time of implantation. Patient 4 was excluded from the rest of the analysis due to the lack of patient-specific dMRI.

**Table 1:** Diagnosis, age, sex, and rating scale scores at baseline, prior to implantation

	Diagnosis	Age at implantation	Sex	HAMD-21 (0–65)	MADRS (0–60)	SHAPS (0–14)	SOFAS (100–0)
Patient 1	MDD	40	Female	28	37	11	30
Patient 2	BD	51	Female	24	29	5	40
Patient 3	MDD	63	Female	14	29	8	30
Patient 4	MDD	47	Female	26	37	12	15

The scale range from no symptoms to severe depression is reported in parenthesis

*HAMD-21* 21-Item Hamilton Depression Rating Scale, *MADRS* Montgomery–Asberg Depression Rating Scale, *SHAPS* Snaith–Hamilton Pleasure Scale, *SOFAS* Social and Occupational Functioning Assessment Scale, *MDD* major depressive disorder, *BD* bipolar disorder with predominant depression

#### Clinical assessments

Patients were routinely assessed after surgery with weekly sessions initially that became less frequent afterwards. Four standardized MDD assessments were adopted: HAMD-21 (21-Item Hamilton Depression Rating Scale) [35], MADRS (Montgomery–Asberg Depression Rating Scale) [36], SHAPS (Snaith–Hamilton Pleasure Scale) [37], and SOFAS (Social and Occupational Functioning Assessment Scale) [38]. The SHAPS scale specifically describes anhedonia and is appropriate to describe the modulation of the NAc area because of this structure’s role in the reward system [39]. The traditionally used HAMD-21 scale is unbalanced towards sleep and circadian dysregulations [40, 41], while the MADRS is considered to be well balanced across the different MDD somatic and cognitive symptoms [42]. Psychiatric assessments were completed by LS, FW, CS, and MS, senior psychiatrists and clinical psychologists with extensive experience and training in assessing psychopathology, including the HAMD and MADRS. They were supervised by SW.

To ease comparison, all scales were normalized onto a scale of 0–100, with high values indicating severe depression. The percentage improvement with respect to baseline was recorded at each clinical assessment and considered as the effect of the most recent DBS parameters applied. The stimulation parameters were fine-tuned to minimize the symptoms and achieve remission (HAMD-21 < 8) or at least response (> 50% improvement of the HAMD-21 score from baseline). The follow-up period was at least 18 months but variable across patients. The number of assessment sessions was 36 for patient 1, 11 for patient 2, and 14 for patient 3, respectively. In each session, the ongoing DBS parameters were recorded.

#### Patient-specific imaging

Pre-implantation neuroimaging was performed on a 3T scanner (Magnetom Skyra Fit, Siemens, Germany) using a 32-channel head receiver coil. The protocol included T1-weighted (TR = 2020 ms, TE = 3.49 ms, voxel resolution 1 × 1 × 1 mm^3^) and T2-weighted (TR = 2400 ms, TE = 225 ms, voxel resolution 1 × 1 × 1 mm^3^) sequences, and a dMRI sequence acquired in the Siemens q-space mode (repetition time = 5900 ms, echo time = 111 ms, voxel resolution 2.2 × 2.2 × 2.2 mm^3^, in-plane acceleration GRAPPA factor of 2, partial Fourier 7/8, field of view 211 × 211 mm^2^). Diffusion weighting, with *b*-values in the range of 0–3000 s/mm^2^ was applied along 123 directions uniformly distributed on the sphere. Postoperative CT or MRI scans were also acquired.

#### Functional imaging

Minimally pre-processed resting-state fMRI acquisitions of 30 random healthy subjects (15 females) from the Human Connectome Project (HCP) database were included to build a reference functional connectome (Supplementary Table 1) [43]. While our clinical sample differed in age and consisted exclusively of females, our random selection of HCP subjects was driven by practical and scientific considerations. The HCP data represents a widely utilized neuroimaging database, including young adults aged 22–35. Furthermore, as TRD can affect both genders, opting for a sex-balanced dataset was justified. Although a larger sample size might have been useful, we limited our sample size to 30 subjects due to computational constraints (analysis took up to 3 days per subject).

The resting-state data consisted of four sessions of approximately 15 min each. Acquisition parameters are described in (https://db.humanconnectome.org). Further pre-processing was performed as in [44], including spatial smoothing and remotion of the first ten volumes, detrending, regressing of nuisance variables (head translation and rotation along the three axes, average cerebrospinal fluid, and white-matter signal) and band-pass filtering [0.01–0.15 Hz]. To avoid well-known artifactual negative correlations induced by global signal regression and the removal of signal of neural origin that might be of interest for the analysis [45, 46], we chose here to be conservative and not perform global signal regression. We obtained a V-by-T data matrix for each subject containing the pre-processed BOLD time-courses, with V the number of voxels and T the number of timepoints. The four sessions were then concatenated temporally to obtain a V-by-4T matrix for each subject [47].

### Lead localization and volume of activated tissue (VAT) estimation

Lead localization was performed with the Lead-DBS toolbox (version 2.5.2) [48] in MATLAB 2020b (The Mathworks, Natick, MA, USA) (Supplementary Fig. 2). Using an early postoperative high-resolution CT or MRI scan, DBS leads were semi-automatically localized either with the PaCER [49] or with the TRAC/CORE [50] algorithm (postoperative CT or MRI case, respectively). For postoperative CTs, the orientation was determined with DiODe [51]. For postoperative MRIs, the orientation was determined with early postoperative X-rays.

**Fig. 1:**
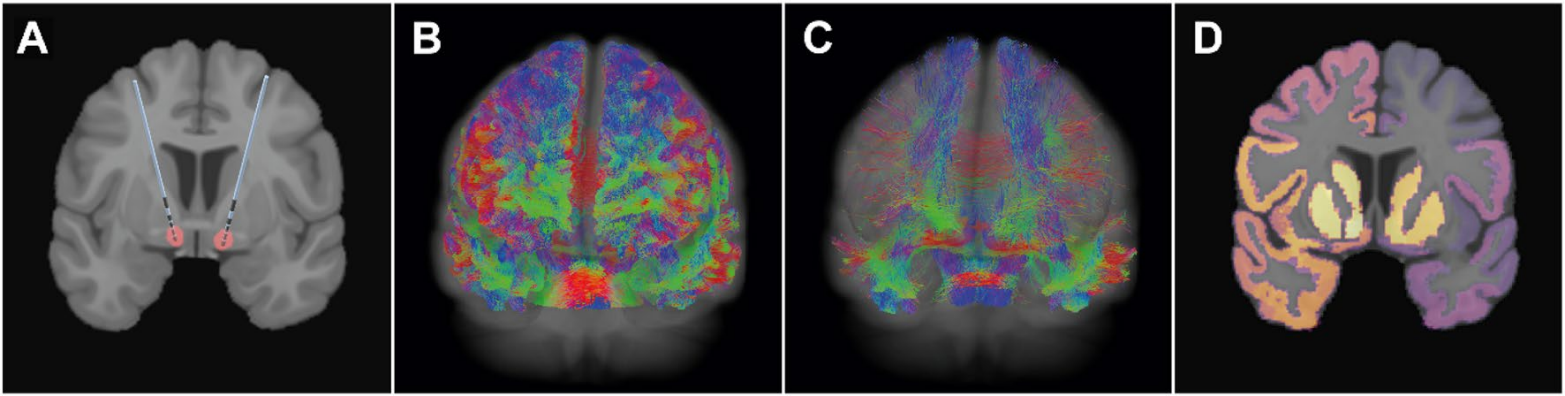
Structural connectivity analysis pipeline. **A** Lead localization in patient space and generation of the bilateral VAT (red volumes) based on individual stimulation parameters. **B** Generation of patient-specific whole-brain tractogram. **C** Selection of streamlines included in the bilateral VAT. **D** Assignment of streamlines’ endpoints to a Brodmann and subcortical parcellation. *VAT* volume of activated tissue

Volumes of Activated Tissue (VATs) were estimated with the SimBio/Fieldtrip pipeline in Lead-DBS with default parameters for the conductivity of gray and white matter and electric field threshold [52]. During the clinical assessments, stimulation parameters for each DBS lead were recorded, including the contact and stimulation intensity. These parameters (Supplementary Table 2) were used to generate the corresponding VATs (Fig. 1A). The VATs were computed for the left and right leads. In total, there were 122 VATs or 61 bilateral VATs.

### DBS connectivity

#### Structural connectivity

The structural connectivity analysis was performed in MRtrix3 (https://www.mrtrix.org/) [53]. Preprocessing steps included: denoising [54], unringing [55], distortion correction with the Synb0-DisCo tool [56], eddy-currents and subject movement correction [57]. To obtain whole-brain probabilistic tractography, we first estimated the individual multi-shell multi-tissue response functions [58]. Based on the population-averaged response functions, we derived the patient-specific fiber orientation distribution through constrained spherical deconvolution [59] and performed intensity normalization [60]. Next, using anatomically constrained tractography [61] we generated 10 million streamlines (Fig. 1B). Finally, by applying the filtering algorithm SIFT2 [62], each streamline got a weight assigned. Since the SIFT2 algorithm considers all generated streamlines, it provides the tractogram with quantitative properties [63] and is, thus, a more accurate metric of structural connectivity than the streamlines’ count [64] or voxel-based structural connectivity approaches [65].

From each patient’s whole-brain tractogram, we selected only the streamlines passing through the bilateral VAT (Fig. 1C). Next, we identified the end points of these streamlines and assigned them to a parcellation containing cortical and subcortical structures (Fig. 1D). Cortical areas were defined with the Broadman parcellation [66], and subcortical areas (including NAc area, amygdala, cerebellum, hippocampus, pallidum, putamen, and thalamus) were defined with FreeSurfer (https://surfer.nmr.mgh.harvard.edu/). Streamlines whose end points were not in any of the defined areas were discarded.

The structural connectivity strength between a VAT and a parcellation area was measured as the sum of the weights of the streamlines that connected the VAT with that area and normalized to the total sum of weights of the streamlines in that VAT. We calculated the structural connectivity strength *profile* for each VAT, as expressed by Eq. (1):

1 $${\text{Structural connectivity strength }}\left( \% \right) _{{{\text{VAT}} \to {\text{area}}_{i} }} = \frac{{\sum {\text{weights of VAT streamlines ending in area}}_{i} }}{{\sum {\text{weights of VAT streamlines assigned in parcellation}}}} \times 100.$$

Finally, the structural connectivity strengths of left and right VATs were combined. As the effect of unilateral stimulation was not evaluated during the assessment sessions, we considered the bilateral VAT from the same session as a single stimulated volume and combined bilateral areas of the parcellation.

#### Functional connectivity

We computed the seed-to-voxel functional connectivity similar to [22, 65]. In particular, we used the fMRI data from 30 Human Connectome Project (HCP) subjects and the patient-specific bilateral VATs as regions of interest. For each clinical assessment and the corresponding bilateral VAT, we performed the following steps to generate the functional connectivity *profile*, in which each voxel represented the functional connectivity strength between the bilateral VAT and the considered target voxel (A).

1. Average the activity across all voxels of a bilateral VAT.
2. Compute the Pearson’s correlation coefficient between a bilateral VAT’s average activity and each non-VAT voxel.
3. Repeat step (2) for each of the 30 HCP subjects.
4. For each voxel, average the Pearson’s correlation coefficient across the 30 HCP subjects.
5. Apply Fisher *z*-transform to obtain a normal distribution of Pearson’s correlation coefficients, to yield a VAT-specific population-averaged connectivity map, what we call functional connectivity *profile.*

**Fig. 2:**
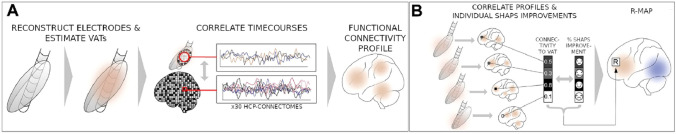
Functional connectivity analysis pipeline. **A** VAT reconstruction and creation of VAT-specific functional connectivity *profile*. Unilateral, rather than bilateral, VAT is shown for simplicity. **B** Creation of a patient-specific *R-map* describing the relation between functional connectivity and symptoms’ improvement. Adapted, with permission, from [22]. *VAT* volume of activated tissue, *HCP* Human Connectome Project

The connectivity *profile* was coupled with the clinical assessment to derive a patient-specific, voxel-wise connectivity *R-map*, as described in the following example with the SHAPS scale (Fig. 2B).

First, we obtained the vector of SHAPS improvement with respect to baseline across clinical assessment sessions for a patient. Since each session corresponds to a functional connectivity *profile*, we considered all the patient’s functional *profiles* and, for each voxel, stored its bilateral VAT connectivity across sessions. The correlation between every voxel connectivity vector and the vector of SHAPS score changes resulted in an *R-map*. The procedure, adopted from [22], was used to obtain an *R-map* for all MDD assessment scales. These personalized *R-maps* are spatial indicators of “optimal” bilateral VAT functional connectivity: positive voxels indicate areas that, when functionally connected to the bilateral VAT, relate to improvement as measured in the corresponding scale. The opposite holds for negative voxels.

### Statistics

For structural connectivity, we obtained structural connectivity strengths, or *profiles*. Then, we computed the Spearman’s correlation coefficients between the connectivity strengths to individual areas in the parcellation and the assessment scores. We repeated the analysis for each MDD scale used. We performed this correlation analysis for the three patients individually. We used false discovery rate-adjusted p-values to determine the statistically significant structural connections. The correlation analysis and false discovery rate corrections were performed in Python (version 3.9.2) with the *scipy* (version 1.9) and *statsmodels* (version 0.13.2) libraries, respectively.

Likewise for functional connectivity, we obtained functional connectivity strengths, or *profiles*. Then we tested their association with clinical outcomes by computing patient-specific *R-maps*. To test the statistical significance of the *R-maps*, we adopted the non-parametric permutation test with custom code in MATLAB 2020b as described in [67] and detailed in the supplementary material.

## Results

### Longitudinal analysis

The follow-up period was at least 18 months in all cases and contained the assessments of the HAMD-21, MADRS, SHAPS, and SOFAS scales. One year after implantation, 2 out of 3 patients were in remission.

**Fig. 3:**
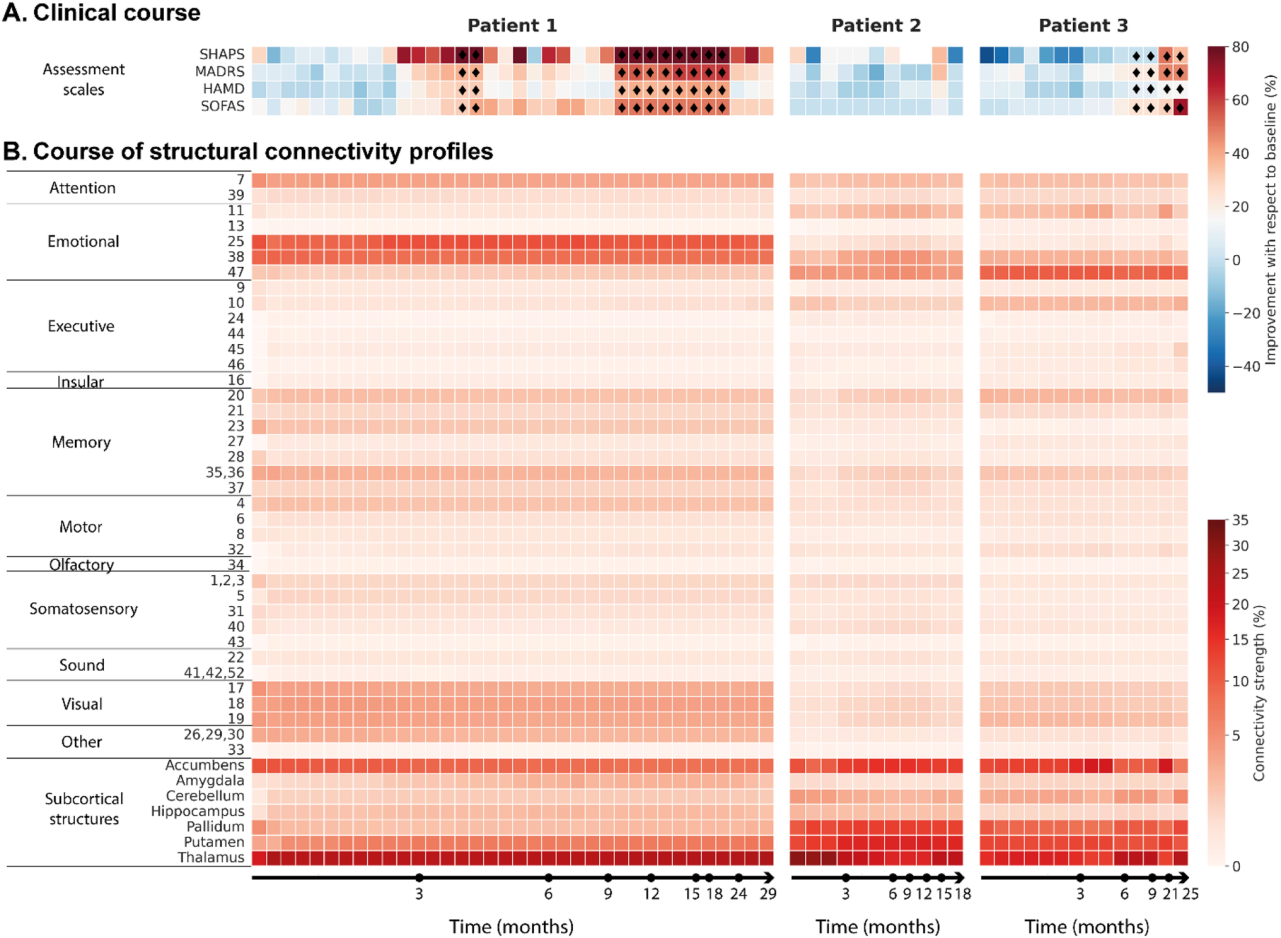
Longitudinal analysis of clinical scales and structural connectivity strengths. **A** Course of clinical rating scale scores. Clinical improvement was obtained as the percentage of improvement with respect to the baseline. *HAMD-21* 21-Item Hamilton Depression Rating Scale, *MADRS* Montgomery–Asberg Depression Rating Scale, *SHAPS* Snaith–Hamilton Pleasure Scale, and *SOFAS* Social and Occupational Functioning Assessment Scale. Remission state is indicated with ⧫. **B** Bilateral VATs structural connectivity *profiles* to Brodmann areas (1–47) and subcortical structures (nucleus accumbens, amygdala, cerebellum, hippocampus, pallidum, putamen, and thalamus). Each column represents the connectivity from a bilateral VAT to the different parcellation areas. Bilateral VATs were estimated from the stimulation settings at each of the assessment sessions. *VAT* volume of activated tissue

For patient 1, an improvement was noticeable at 2.5 months post-implantation (session 10), especially on the SHAPS scale. From 9 months post-implantation (session 25), the improvement was observed across all scales, corresponding to a prolonged remission period of 10 months. In the last three sessions, a worsening on the HAMD scale occurred, marking the end of remission (Fig. 3A).

For patients 2 and 3, a similarly strong improvement across scales was not observed. But for these patients the number of assessments was lower, covering a period of 19 and 26 months, respectively. For patient 3, a remission period of 16 months started at 9 months post-implantation (session 11), with improvements on the MADRS and SOFAS scales. Improvements in the SHAPS scales occurred from month 22 post-implantation (session 13).

### Structural connectivity

First, we analyzed the structural connectivity over the follow-up period. The three patients’ structural connectivity *profiles* from the bilateral VATs to the Brodmann Areas (BAs) shared, with different intensities, strong cortical connections to emotional (BA 11, 38, 47), attentional (BA 7), visual (BA 17–19), memory (BA 20, 35, 36, 37) and executive (BA 10) areas (Fig. 3B). In patient 1, we observed a strong connectivity to the subgenual anterior cingulate cortex (BA 25) and the temporopolar area (BA 38). The connectivity to these areas was stable across the bilateral VATs, with a median value of 10.4% (Inter-quartile range, IQR 9.8–11.5) for BA 25 and 8.2% (IQR 8.1–8.3) for BA 38. In patients 2 and 3, a strong connectivity to BA 25 was not observed, but the median connectivity to BA 38 was 3.3% (IQR 2.7–4.3) and 2.7% (IQR 2.3–2.7), respectively (Fig. 3B). In these two patients, we also observed enhanced connectivity to the prefrontal cortex (BA 10) and orbitofrontal area (BA11) compared to patient 1, but less than for the previously mentioned areas.

Structural connectivity to the subcortical structures was also relevant (Fig. 3B). The three patients shared a strong connectivity to the thalamus, putamen and accumbens area, while patients 2 and 3 also had a strong connectivity to the pallidum. The median thalamic connectivity of patient 1 (23.2%, IQR 22.9–24) was more stable across the bilateral VATs and stronger than for patient 2 (22.2%, IQR 17.9–25.6) or patient 3 (17.2%, IQR 15.5–21.2).

**Fig. 4:**
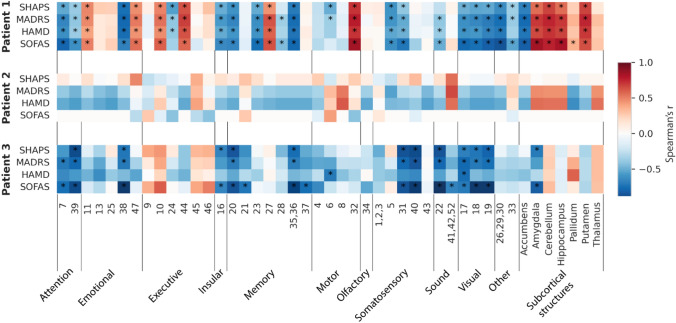
Spearman’s correlations between the assessment scores and the structural connectivity strength from the bilateral VATs to the parcellation areas. Correlation coefficients were calculated for each patient individually. Significant correlations after correcting for type I errors with false discovery rate are marked with an asterisk. Brodmann areas in the parcellation (1–47) are grouped into functional areas (e.g., somatosensory, motor, executive, emotional, insular, attention, memory, visual, olfactory, sound). *VAT* volume of activated tissue

Second, we analyzed correlations between the structural connectivity and the clinical outcome. In patient 1, structural connectivity to the motor (BA 32), executive (BA 10 and 44), and emotional (BA 11 and 47) cortices was positively correlated with improved outcomes (Fig. 4). Connectivity to the amygdala, cerebellum, hippocampus, and putamen was also positively correlated with improved outcomes. In contrast, in patients 2 and 3, the structural connectivity was not significantly correlated with outcome (Fig. 4).

### Functional connectivity

**Fig. 5:**
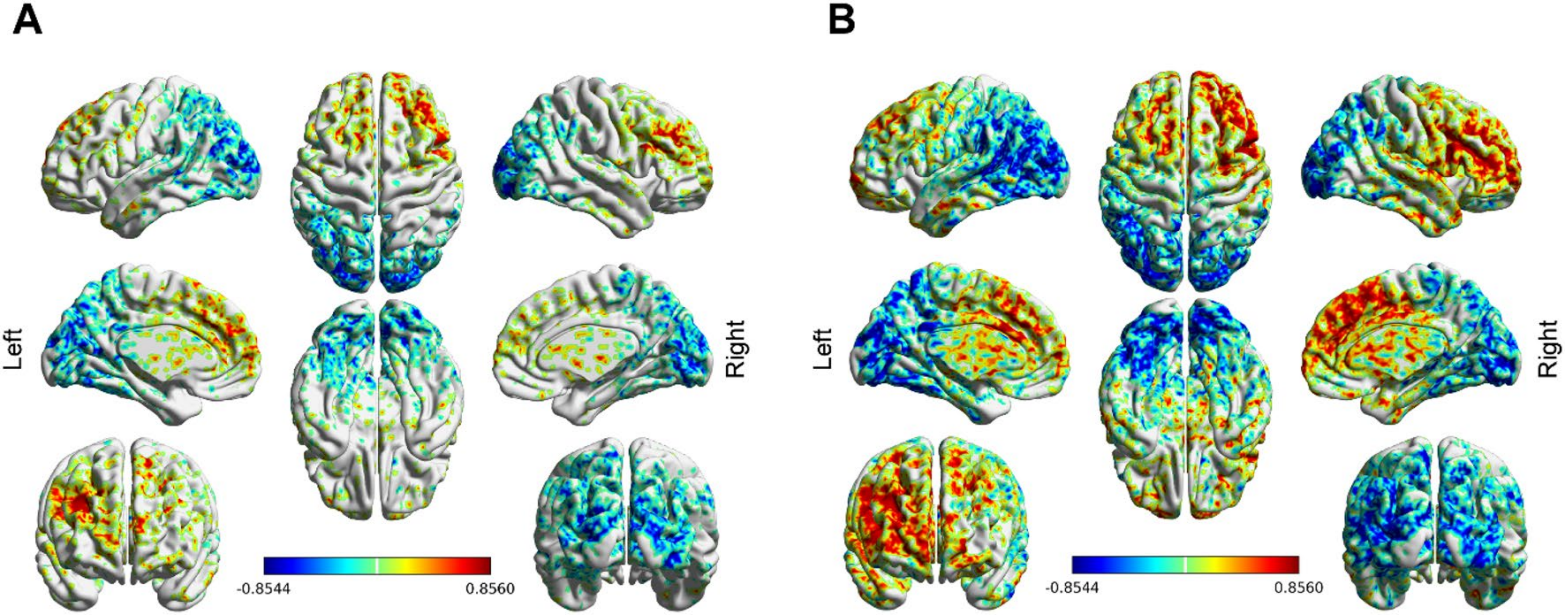
Functional connectivity *R-maps* of patient 1, visualized with BrainNet Viewer [88]. **A** SHAPS (Snaith–Hamilton Pleasure Scale); **B** MADRS (Montgomery–Asberg Depression Rating Scale)

For patient 1, some areas demonstrated significant correlations between functional connectivity and symptoms improvement (Fig. 5). The red areas in Fig. 5, corresponding mainly to executive areas (BA 9 and 10), were correlated with improvement on the SHAPS and MADRS scales. Conversely, blue areas were negatively correlated to improvement on these two scales. These mainly corresponded to visual (BA 18 and 19) and attentional areas (BA 7). Across scales, the areas of positive and negative correlation to improvement were similar in location, but the size of the areas varied.

The in-depth voxel-wise analysis of the significant *R-maps* is shown in Supplementary Tables 3 and 4. The functional connectivity analyses of patients 2 and 3 were non-significant. For these patients, no brain area was significantly correlated with improvement on any of the scales.

## Discussion

In this retrospective case series of TRD patients implanted with DBS in the NAc, no serious adverse events were noted. The structural and functional connectivity *profiles* of the VATs were mixed across patients.

### Structural connectivity

Our study highlighted a structural connectivity of the bilateral VATs to the subgenual anterior cingulate cortex (BA 25) in patient 1, who had the longest remission period. This connection was expected from both dissection [68] and tractography studies [69]. BA 25 has been implicated in persistent sad mood [70], circadian dysregulations [13] and rumination [71] and is a key node of the affective network [6]. This area is metabolically hyperactive in MDD patients [72], and successful treatment through medication (e.g., fluoxetine, venlafaxine), electro-convulsive therapy, cognitive behavioral therapy, and repetitive transcranial magnetic stimulation has been associated with a reduction in its activity [73, 74]. Interestingly, for patients 2 and 3, the structural connectivity to BA 25 was less prominent and coincided with less clinical improvements.

As in [13, 70, 75], we hypothesized a correlation between this area’s connectivity to the bilateral VATs and clinical improvement. In patient 1, the structural connectivity was strongest to BA 25 and it was stable across sessions. In this subject, structural connectivity to this area was positively correlated to the outcome, but was statistically non-significant. The limited intrasubject changes in BA 25 connectivity could derive from the very few changes in the DBS contact selection and from the time-invariance assumption of the whole-brain tractogram (i.e., assuming the tractogram does not change with implantation nor over time).

Riva-Posse et al. [76] identified four bundles whose activation was correlated with clinical response to DBS in BA 25 for TRD: bilateral forceps minor, bilateral cingulum bundles, dorsal and medial midcingulate cortices, and uncinate fasciculus. The uncinate fasciculus interconnects the lateral orbitofrontal cortex and BA10 to the limbic regions of the anterior temporal lobes. The structural connectivity of our best responder (patient 1) also highlighted connection to the temporopolar area (BA 38). The structural connectivity between our target site (i.e., NAc area) and the temporopolar area suggests activation of the uncinate fasciculus bundle, thus agreeing with [76]. The structural connectivity *profiles* of patients 2 and 3 also showed connectivity to BA 38; but in the correlation analysis, connectivity to this area was not positively correlated with clinical improvement.

GABA-ergic and dopaminergic connections between the NAc, amygdala, and hippocampus seem to be related to the regulation of emotional responses [27, 77]. In our best responder, patient 1, we observed a higher structural connectivity to the amygdala and hippocampus; and in the correlation analysis, these areas were positively correlated with the outcome. This finding in patient 1 is in line with a recent DBS study using network mapping and biomarker discovery, which also highlighted the connectivity to the amygdala as an indicator of symptom improvement [77].

### Functional connectivity

The optimal connectivity maps obtained allowed us to relate the bilateral VATs’ functional connectivity to the outcomes. The cortical areas most strongly correlated to a positive outcome were all belonging to the prefrontal cortex (BA 8, 9, 10, 24, 32, 45, 46). The prefrontal cortex is thought to give rise to the psychomotor and cognitive aspects of TRD [78, 79] and is a commonly used repetitive transcranial magnetic stimulation target for TRD [80]. In the functional connectivity maps, BA 25 either showed an average correlation coefficient close to zero or had a low number of significant voxels. This agrees with our structural connectivity analysis that BA 25 is structurally connected to the bilateral VATs irrespective of the outcome. For patients 2 and 3, there was no significant voxel after the permutation test. This might have a twofold explanation. First, these patients only had 11 and 14 assessment sessions, respectively, while patient 1 had 36. Besides, the sessions of patients 2 and 3 did not show a large variability in outcomes. Therefore, the creation of functional connectivity maps may crucially depend on the availability of larger and more variable patient outcome data.

### Nucleus accumbens area as a DBS target

Our study expands the literature on the NAc as DBS target. The NAc seems to be at the center of a limbic corticostriatal loop, between the emotional system, the cognitive system and the motor control system [74]. Given the area’s cortical and subcortical connections, we included both in the parcellation used to study the structural connectivity *profiles* of DBS. Because of its role in the reward network [81], the NAc is an established DBS target for TRD and it is especially suited for depression types with prevailing anhedonia [25].

Our response rate was in line with the open-label NAc DBS studies of Bewernick et al. [33, 34], which reported a 50% response rate and a 30% remission rate at 1-year follow-up, and 45.5% and 9.1% at 2-year follow-up. These response rates were comparable to those of other DBS targets for TRD such as the medial forebrain bundle [82, 83], the subgenual anterior cingulate cortex target [84, 85], and the inferior thalamic peduncle [86, 87].

The improvements observed in our best responder may have a twofold explanation. On the one hand, there was structural and functional connectivity from the VATs to nodes of the depression network such as BA 25, the thalamus, amygdala, or the prefrontal cortex. On the other hand, the cortical connectivity of our target site suggests a possible overlap with the medial forebrain bundle or ventral tegmental area projection pathway, which has been proposed as a DBS target [88]. The tract is highly similar to the NAc crossing bundle described by [23], which has been related to improvement for obsessive–compulsive disorder, a condition sharing many features with MDD. Since single-target clinical studies failed to prove efficacy, we might argue, as in [19, 76, 89], that the stimulation of a unique target is insufficient for remission, and future protocols should aim to engage the distributed depression network instead.

### Study limitations

The present work has several limitations. First, it is restricted to three subjects, some assessed in few sessions. The study was open label and did not have standardized time intervals during long-term follow-ups. The stimulation parameters were changed based on the previous symptom’s evolution in a trial-and-error fashion, potentially introducing a placebo effect. However, typically, the placebo effects are less pronounced in subjects with severe or chronic depression [90].

Second, our analysis did not consider medications or external life events. These are likely to affect the clinical outcome but may not be reflected in the connectivity *profiles* or *R-maps*. We acknowledge that the assumption of DBS as the unique or main source of clinical outcome is a simplification.

Lastly, we used fMRI data from the Human Connectome Project, rather than patient-specific fMRI, to create functional connectivity *R-maps*. Normative connectomes have been leveraged for the study of many psychological conditions. In the DBS field, the use of normative data has been a key to identify patterns of beneficial connectivity [22, 65]. While the patient data should be more accurate in describing pathological connections, a recent study suggests that the difference is minimal and counterbalanced by the quality of large datasets such as the Human Connectome Project [91].

### Conclusions and outlook

We have leveraged patient-specific diffusion images and probabilistic tractography to quantify the structural connectivity of NAc area DBS to cortical and subcortical brain areas. The subgenual anterior cingulate cortex (BA 25), known for its involvement in depression, was consistently and strongly connected to bilateral VATs in the patient experiencing extended periods of remission. This suggests that effective NAc area DBS for TRD may modulate a network shared also with other DBS targets.

We created statistically significant *R-maps* associating the bilateral VATs’ functional connectivity and improvements. Their analysis revealed that connectivity to the prefrontal cortex was associated with improvement in one patient. Once validated with more MRI data and statistical tests, these *maps* could have important clinical applications and could suggest stimulation parameters, thus becoming valuable features for personalized closed-loop DBS [92]. Furthermore, they could guide complementary TRD therapies that would only engage areas linked to improvement.

Our work adopts the much needed [19] patient-centric approach in the description of DBS connectivity. Overall, the results were variable among the three patients and could only partially describe the observed clinical TRD progression. Further studies, including more patients over longer time periods, are needed to better understand the connectivity of effective DBS for TRD. In particular, we encourage prospective NAc DBS studies to adopt blinded multidimensional assessment of MDD symptoms, including longitudinal ambulatory assessments using ecological momentary assessments, and to include other neuroimaging data, e.g., fMRI and EEG, repeated over multiple sessions.

### Supplementary Information

Below is the link to the electronic supplementary material.

Supplementary file1 (DOCX 1115 KB)

## Data Availability

Pipelines for dMRI processing and data analysis are available in: https://gitlab.switch.ch/brain-stimulation-mapping/2025_dbsinmdd. This is a preliminary repository, and the name will be updated upon publication. A de-identified data set containing tracts in patient space, the MNI VTAs and transformations from MNI to patient space can be provided on application, subject to institutional review board approval.
