# Supplementary material for: Linking connectivity of deep brain stimulation of nucleus accumbens area with clinical depression improvements: a retrospective longitudinal case series
Source: Eur Arch Psychiatry Clin Neurosci. 2023 Sep 5;274(3):685–96. doi: 10.1007/s00406-023-01683-x (PMC10994999; doi:10.1007/s00406-023-01683-x)
Supplement: Supplementary file 1 — Supplementary file1 (DOCX 1115 KB) [file 406_2023_1683_MOESM1_ESM.docx]

# Supplementary materials

## Functional MRI data

Table S1 : Demographics of the HCP subjects used in the functional analysis. HCP, Human Connectome Project

| Subject ID | Release | Acquisition | Gender | Age |
| --- | --- | --- | --- | --- |
| 100307 | Q1 | Q01 | F | 26-30 |
| 100408 | Q3 | Q03 | M | 31-35 |
| 101107 | S500 | Q06 | M | 22-25 |
| 101309 | S500 | Q06 | M | 26-30 |
| 101915 | Q3 | Q04 | F | 31-35 |
| 103111 | S500 | Q06 | M | 26-30 |
| 103414 | Q2 | Q02 | F | 22-25 |
| 103818 | Q1 | Q01 | F | 31-35 |
| 105014 | S500 | Q05 | F | 26-30 |
| 105115 | Q2 | Q02 | M | 31-35 |
| 106016 | Q3 | Q04 | F | 31-35 |
| 108828 | S500 | Q05 | M | 31-35 |
| 110411 | Q2 | Q02 | M | 31-35 |
| 111312 | Q1 | Q01 | F | 31-35 |
| 111716 | Q3 | Q04 | F | 31-35 |
| 113619 | Q2 | Q02 | F | 31-35 |
| 113922 | S500 | Q04 | M | 31-35 |
| 114419 | S500 | Q07 | M | 31-35 |
| 115320 | Q2 | Q02 | F | 31-35 |
| 116524 | S500 | Q05 | M | 26-30 |
| 117122 | Q1 | Q01 | F | 26-30 |
| 118528 | S500 | Q04 | F | 26-30 |
| 118730 | Q2 | Q03 | M | 22-25 |
| 118932 | Q1 | Q02 | M | 26-30 |
| 120111 | Q3 | Q03 | F | 26-30 |
| 122317 | Q3 | Q04 | M | 31-35 |
| 122620 | S500 | Q05 | M | 26-30 |
| 123117 | Q2 | Q03 | M | 26-30 |
| 123925 | S500 | Q05 | F | 26-30 |
| 124422 | Q2 | Q03 | F | 31-35 |

## Permutation test

The permutation test used adopted to test the significance of the functional connectivity R-map had the following null hypothesis:

*H_0_: In each subject, the same clinical improvement would arise independently of the voxel-to-VAT connectivity. Equivalently, the labeling of the voxels by the corresponding empirical clinical improvement would be arbitrary and any statistic’s distribution would be unaltered by any permutation of the labels.*

The assumed absence of any confound on the labeling attribution led to the full exchangeability of the labels. We considered 99 of the #sessions! Possible permutations, thus performing a conservative approximate test. For each permutation, we obtained the maximal and minimal R-statistic. From the distribution of the maximal (and minimal) statistics, we derived a threshold for the empirical R-map defined as the c + 1 biggest (lowest) permutation distribution member, where c = floor(α ∗ N), α = 0.01 and N = number of permutations + 1 (the actual labeling). The null hypothesis was rejected for any voxel with a statistic value greater (or smaller) than the maximal (or minimal) critical threshold. Since this critical value was computed by considering the extreme values of the statistic image over all the permutations, the single threshold test adopted also accounted for the multiple comparison problem (1). Of note, the single threshold test is known to cause sensitivity loss in voxels with lower variability (1). To counterbalance such sensitivity loss, we initially smoothed the fMRI data and we Fisher z-transformed the functional connectivity fingerprints.

Lastly, we used the Brodmann parcellation (2) to identify the location of the significant voxels and determine, for each BA: the average R value of significant voxels, the percentage of significant voxels, and the proportion of significant cortical voxels belonging to that area over all cortical voxels.

## Lead localization


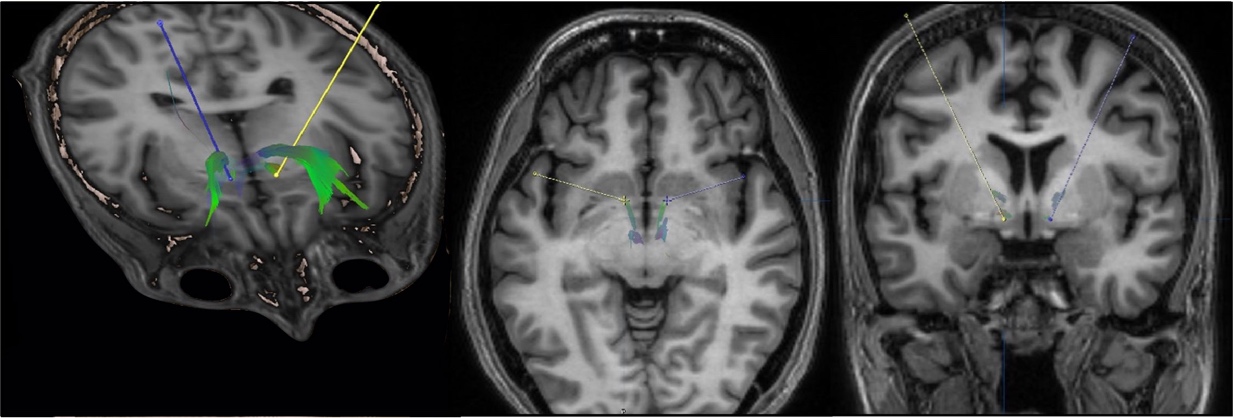


Figure S1. DBS targeting of the NAc area in one patient. The target was placed just posterior to the NAc in order to include the afferent fibers of the medial forebrain bundle (green streamlines) before reaching the anterior limb of the internal capsule. DBS, Deep Brain Stimulation; NAc, nucleus accumbens.


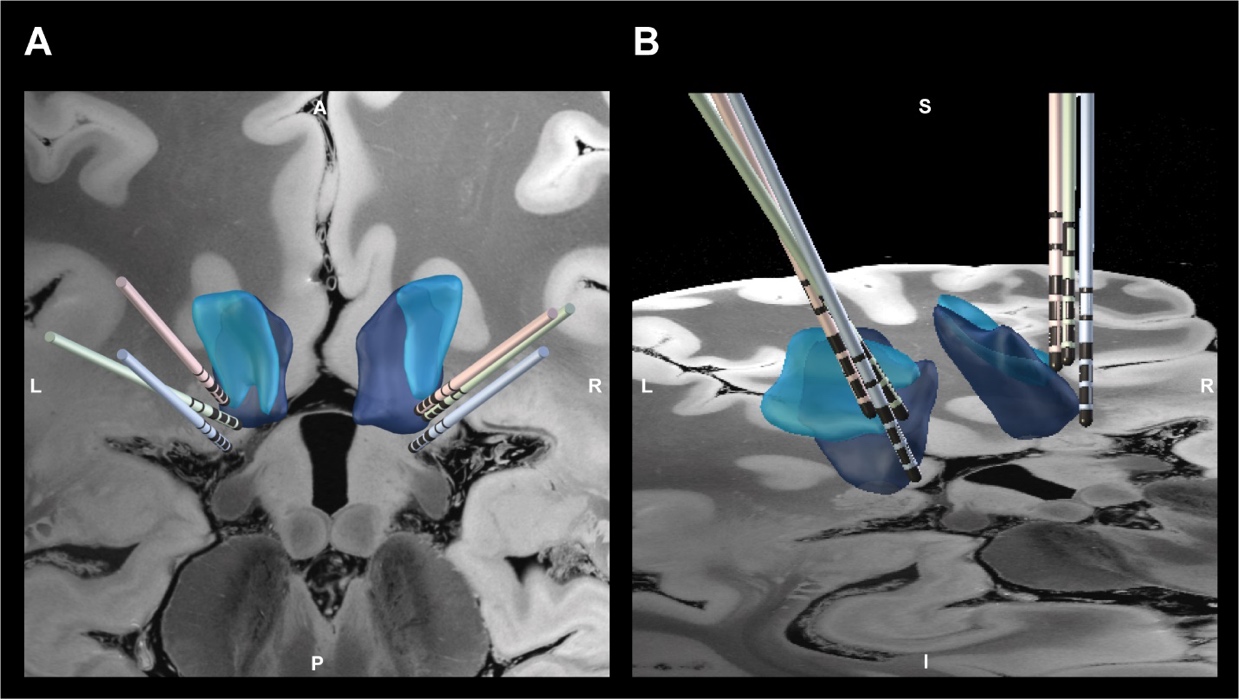


Figure S2: Lead localization in MNI space. A: Superior axial view. B: Rotated axial view. Patient 1: blue leads, Patient 2: red leads, Patient 3: green leads. NAc core in navy blue, NAc shell in cyan.NAc, nucleus accumbens.

## DBS parameters

Table S2. Stimulation intensities (mA) for left and right hemispheres for each patient and session.

## R-maps analysis

Table S3: Patient 1 SHAPS R-map analysis, sorted by share of significant voxels

| Brodmann Area | Average R value over significant voxels | Perthousand survived voxels in the area | % significant voxels |
| --- | --- | --- | --- |
| BA19 | -0.737 | 1.11 | 12.42 |
| BA18 | -0.742 | 0.85 | 9.49 |
| BA9 | 0.744 | 0.85 | 9.46 |
| BA7 | -0.718 | 0.62 | 6.92 |
| BA17 | -0.743 | 0.57 | 6.4 |
| BA8 | 0.726 | 0.54 | 6.05 |
| BA10 | 0.717 | 0.5 | 5.61 |
| BA6 | 0.673 | 0.49 | 5.46 |
| BA39 | -0.739 | 0.48 | 5.4 |
| BA37 | -0.706 | 0.37 | 4.17 |
| BA40 | -0.64 | 0.31 | 3.52 |
| BA31 | -0.603 | 0.26 | 2.92 |
| BA4 | 0.463 | 0.22 | 2.49 |
| BA32 | 0.706 | 0.19 | 2.17 |
| BA22 | -0.286 | 0.15 | 1.75 |
| BA45 | 0.736 | 0.14 | 1.58 |
| BA46 | 0.72 | 0.13 | 1.55 |
| BA44 | 0.591 | 0.12 | 1.4 |
| BA21 | 0.025 | 0.12 | 1.34 |
| BA23 | -0.711 | 0.1 | 1.22 |
| BA24 | 0.745 | 0.07 | 0.82 |
| BA2 | -0.28 | 0.07 | 0.8 |
| BA35_36 | -0.35 | 0.06 | 0.7 |
| BA47 | 0.647 | 0.06 | 0.7 |
| BA5 | -0.678 | 0.06 | 0.68 |
| BA1_3 | 0.259 | 0.06 | 0.68 |
| BA20 | 0.264 | 0.05 | 0.62 |
| BA28 | -0.645 | 0.05 | 0.59 |
| BA38 | 0.616 | 0.04 | 0.55 |
| BA43 | 0.347 | 0.04 | 0.5 |
| BA26_29_30 | -0.734 | 0.04 | 0.49 |
| BA41_42_52 | -0.67 | 0.04 | 0.48 |
| BA16 | 0.133 | 0.04 | 0.47 |
| BA13 | 0.642 | 0.01 | 0.16 |
| BA11 | 0.289 | 0.01 | 0.16 |
| BA33 | 0.746 | 0.01 | 0.12 |
| BA27 | -0.571 | 0 | 0.11 |
| BA25 | 0.37 | 0 | 0.05 |
| BA34 | 0 | 0 | 0 |

Table S4: Patient 1 MADRS R-map analysis, sorted by share of significant voxels

| Brodmann Area | Average R value over significant voxels | Perthousand survived voxels in the area | % significant voxels |
| --- | --- | --- | --- |
| BA6 | 0.583 | 1.97 | 8.48 |
| BA19 | -0.692 | 1.89 | 8.14 |
| BA9 | 0.682 | 1.5 | 6.48 |
| BA8 | 0.703 | 1.45 | 6.28 |
| BA10 | 0.625 | 1.29 | 5.58 |
| BA37 | -0.562 | 1.26 | 5.46 |
| BA18 | -0.713 | 1.24 | 5.36 |
| BA40 | -0.357 | 1.18 | 5.08 |
| BA7 | -0.653 | 1.08 | 4.68 |
| BA22 | -0.151 | 0.88 | 3.8 |
| BA39 | -0.707 | 0.86 | 3.73 |
| BA17 | -0.716 | 0.83 | 3.6 |
| BA4 | 0.369 | 0.74 | 3.2 |
| BA21 | -0.033 | 0.63 | 2.72 |
| BA32 | 0.669 | 0.55 | 2.39 |
| BA31 | -0.327 | 0.54 | 2.37 |
| BA44 | 0.531 | 0.49 | 2.13 |
| BA45 | 0.621 | 0.44 | 1.92 |
| BA47 | 0.517 | 0.4 | 1.75 |
| BA38 | 0.298 | 0.35 | 1.53 |
| BA24 | 0.701 | 0.35 | 1.52 |
| BA20 | 0.122 | 0.34 | 1.5 |
| BA46 | 0.625 | 0.31 | 1.35 |
| BA1_3 | 0.145 | 0.31 | 1.35 |
| BA2 | -0.063 | 0.28 | 1.23 |
| BA35_36 | -0.327 | 0.25 | 1.11 |
| BA23 | -0.323 | 0.23 | 1.02 |
| BA43 | 0.535 | 0.21 | 0.93 |
| BA16 | 0.113 | 0.21 | 0.92 |
| BA28 | -0.338 | 0.18 | 0.79 |
| BA41_42_52 | -0.427 | 0.17 | 0.76 |
| BA13 | 0.567 | 0.15 | 0.66 |
| BA5 | -0.502 | 0.14 | 0.61 |
| BA11 | 0.393 | 0.11 | 0.49 |
| BA26_29_30 | -0.623 | 0.08 | 0.36 |
| BA27 | 0.117 | 0.06 | 0.28 |
| BA25 | 0.431 | 0.04 | 0.21 |
| BA34 | 0.348 | 0.02 | 0.12 |
| BA33 | 0.727 | 0.02 | 0.1 |


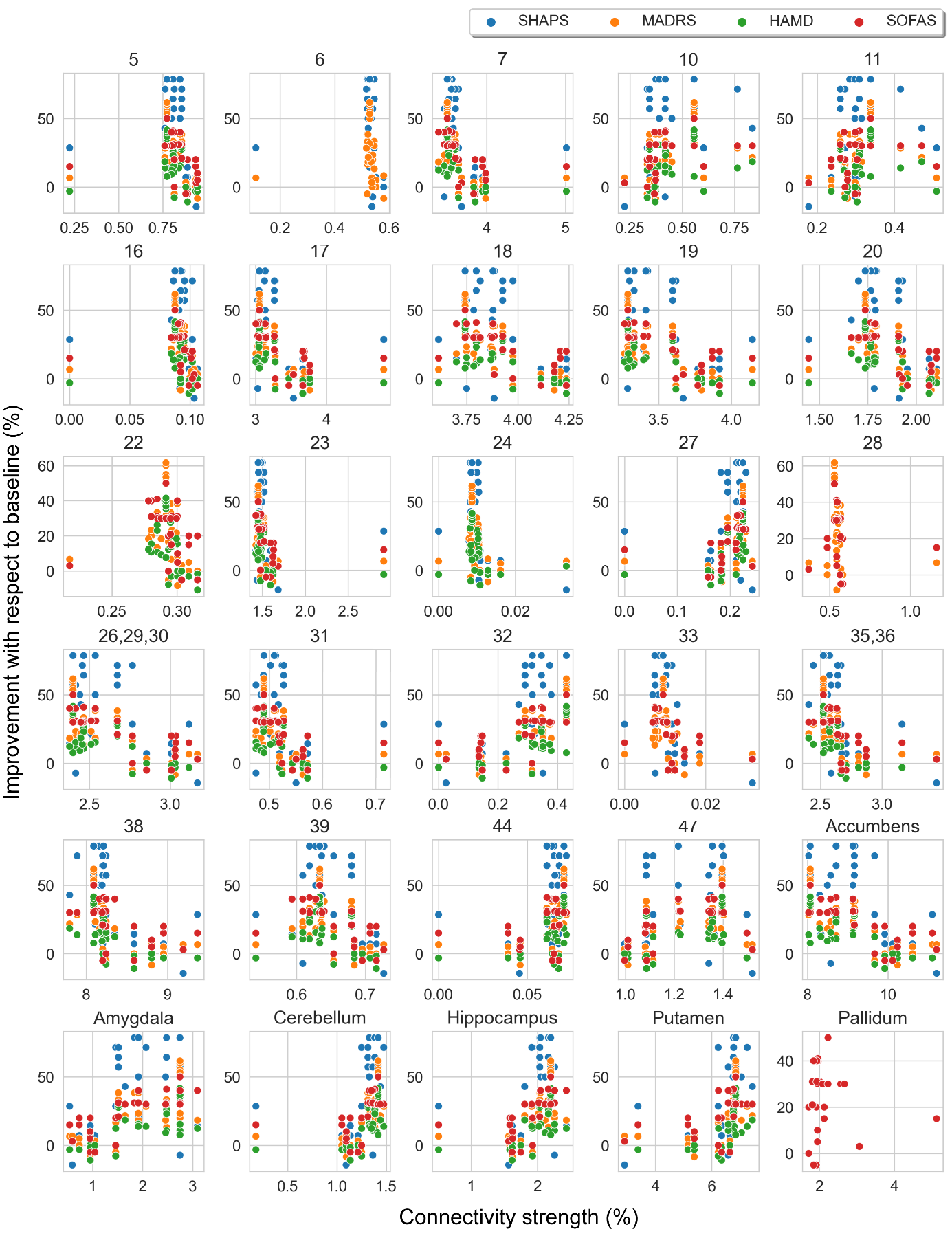


Figure S2. Scatterplots for the significant correlation results of the structural connectivity analysis in Patient 1.


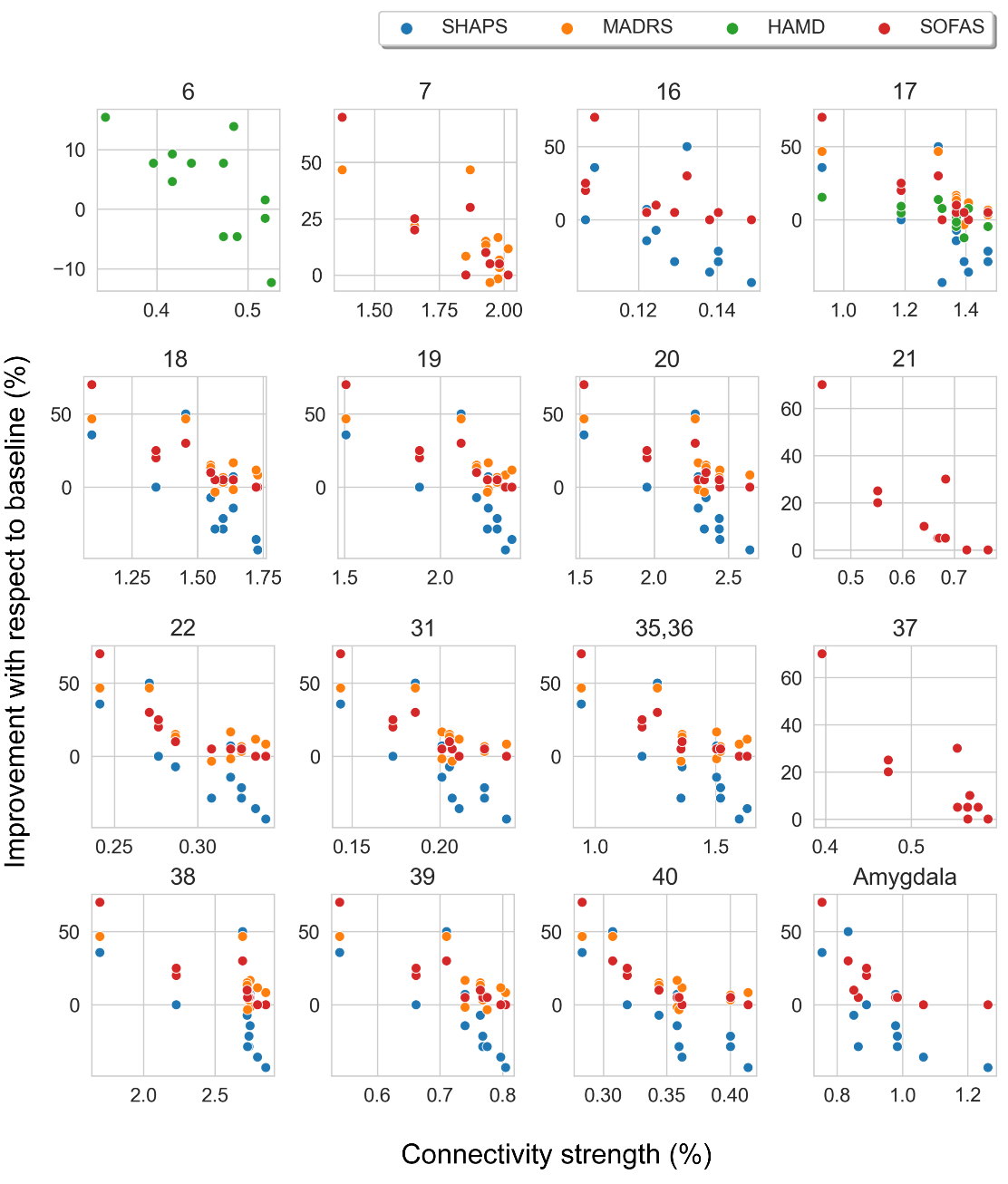


Figure S3. Scatterplots for the significant correlation results of the structural connectivity analysis in Patient 3.

## References

1. Nichols TE, Holmes AP (2002): Nonparametric permutation tests for functional neuroimaging: a primer with examples. *Hum Brain Mapp* 15: 1–25.

2. Pijnenburg R, Scholtens LH, Ardesch DJ, de Lange SC, Wei Y, van den Heuvel MP (2021): Myelo-and cytoarchitectonic microstructural and functional human cortical atlases reconstructed in common MRI space. *NeuroImage* 239: 118274.
